# The congested International Match Calendar in football: views of 1055 professional male players

**DOI:** 10.1186/s13102-022-00597-w

**Published:** 2022-11-29

**Authors:** L. Pillay, D. Burgess, D. C. Janse van Rensburg, G. M. Kerkhoffs, V. Gouttebarge

**Affiliations:** 1 Amsterdam UMC location University of Amsterdam, Department of Orthopedic Surgery and Sports Medicine, Meibergdreef 9, 1105 AZ Amsterdam, The Netherlands; 2 Section Sports Medicine, University of Pretoria, Pretoria, South Africa; 3 Adelaide Football Club, Adelaide, Australia; 4 Amsterdam Collaboration on Health and Safety in Sports (ACHSS), IOC Research Center of Excellence, Amsterdam, The Netherlands; 5 Academic Center for Evidence Based Sports Medicine (ACES), Amsterdam, The Netherlands; 6 Amsterdam Movement Sciences, Aging & Vitality, Musculoskeletal Health, Sports, Amsterdam, The Netherlands; 7 Football Players Worldwide (FIFPRO), Hoofddorp, The Netherlands

**Keywords:** Football, Congestion, Performance, Recovery, International

## Abstract

**Background:**

The International Match Calendar congestion affects players recovery. The views of a worldwide cohort of professional football players is shared in this communication.

**Methods:**

A cross-sectional observational study recruited players through Fédération Internationale des Associations de Footballeurs Professionnel’s national members. An electronic survey was shared in English, French, Italian and Spanish with 1055 players consenting and completing it anonymously in November 2021.

**Results:**

A total of 42% of respondents believe back-to-back matches should be limited to three. Most respondents (69%) felt off or in season breaks are infringed by clubs or national teams and 83% believe regulations should protect them. A total of 55% of players believed they sustained one or more injuries due to the overload and it has affected 52% of respondents’ mental state.

**Conclusion:**

The congested International Match Calendar poses a risk to professional footballers physical and mental health. Poor recovery between matches may affect player availability and performance. Players should be represented by active players when International Match Calendar scheduling decisions are made. Administrators should seek medical guidance regarding the effects of overload on performance prior to making decisions. This study allows the opportunity for a larger national team player sample to be studied.

## Introduction

The International Match Calendar (IMC) determines the dates for all international related football matches and competitions (be it national team or club competitions e.g. Federation Internationale de Football Association World Cup (FIFA) qualifiers, Union of European Football Associations (UEFA) Champions League). Administrators principally decide upon the dates at FIFA. The IMC provides professional footballers with little or no time to rest and recover after repetitive high workloads. The Fédération Internationale des Associations de Footballeurs Professionnels (FIFPRO) is a worldwide representative for professional footballers. In 2019, FIFPRO showed increased physical and mental demands on professional footballers due to the IMC, especially for those combining domestic and international competitions [1]. Recently, the coronavirus (COVID-19) pandemic has further contributed to match congestion. Soon after the World Health Organisation (WHO) declared COVID-19 a global outbreak [2], football leagues around the world (including international football) cancelled matches due to the unknown risks that existed regarding epidemiology, pathogenesis [3, 4] and related complications [5]. However, teams had to still fulfil fixtures to ensure the continuity of the industry [6]. Due to the IMC and associated match congestion, professional footballers have a high risk for injuries, with a mean time-loss injury rate reaching up to 27.5 injuries per 1000 match hours [7–9]. Acute to chronic workload ratio has been shown as an important risk factor for non-contact related injuries [10].

The combination of all the above has significantly strained the professional football player—physically and mentally [6, 11]. Furthermore, FIFA has recently proposed a World Cup event to happen every two years instead of four—this would further increase workload and compromise players’ health. Subsequently, leaving less time to implement active and passive recovery methods between higher workloads from matches, training, and travel and fewer off-season periods. This will ultimately affect players’ performances as well as their physical and mental health [6, 11–14].

In order to safeguard the performances and health of professional footballers, there is a need to explore players’ views on how the IMC presently affects them. Consequently, we aimed to explore the view of professional footballers on various elements related to the IMC, namely workload, recovery, mental well being and international duty. This study builds upon the study by Goutterbarge, Brink and Kerkhoffs(2019) (The perceptions of elite professional footballers on the International Match Calendar: a cross-sectional study), where only 543 FIFPRO registered professional football players were involved and only five aspects were explored—(1) playing too many matches per season, (2) negative impact on number of matches, (3) insufficient recovery between matches, (4) negative impact of long-distance flights and (5) in-season and off-season breaks. The current study involved 1055 players and apart from questions related to participant characteristics, (1) there were more respondents from other continental divisions apart from Europe, (2) more players' views concerning match load, (3) more players views regarding regulations, fixture schedules, and it’s effect on physical and mental health and (4) collaboration between club and country medical staff.

## Methods

### Design

An observational study based on a cross-sectional design using a survey was conducted. Ethical approval for the study was provided by the Medical Ethics Review Committee of the Amsterdam University Medical Centers (Amsterdam UMC, location AMC; W21_493 # 21.548). All methods were performed in accordance with the relevant guidelines and regulations.

### Participants

The study population consisted of male professional footballers recruited by FIFPRO and its national members. The data collection period started during the resumption of international football in the COVID-19 pandemic. Most leagues and international competitions that resumed during this time, involved male participants. Inclusion criteria were: (1) a professional footballer, (2) age 18 or older, (3) male and (4) able to read and comprehend texts fluently in English, French, Italian or Spanish. For this study, the definition for a professional footballer was that he (i) trains to improve performances, (ii) competes in the highest or second highest national league and (iii) has football training and competition as major activity (way of living) or focus of personal interest, devoting several hours in all or most of the days for these activities and exceeding the time allocated to other types of professional or leisure activities. The cohort involves players that play for their National teams and those that do not. The reason for this is because some competitions that the IMC dictates are club-based competitions (e.g. Confederation of African Football [CAF] Champions League). FIFPRO comprises of ± 65,000 members worldwide. Due to the observational nature of our study, the sample size calculated using http://www.raosoft.com/samplesize.html [15] was n = 245 (Confidence interval = 95% and response distribution of 20%) for statistical significance.

### Survey

Experts from football stakeholders (governing bodies, players’ unions, current/retired players) gathered in September 2021 to define questions related to workload in men’s professional football. These questions were divided into three distinct sections, namely (1) characteristics based on six questions, e.g., “For how many years have you been playing men’s professional football?”, (2) workload, recovery and well-being based on seven questions, e.g., “What is the maximum number of back-to-back matches a player should play consecutively?” Back-to-back matches are defined by Klynfeld Peat Marwick Goerdeler [KPMG] Football Benchmark as a player being on the pitch for at least 45 min after playing a minimum of 45 min in the previous game—with less than 5 days of recovery time between the two appearances) and (3) international football based on four questions,e.g., “How often should the FIFA World Cup Finals take place?”. Questions were answered on different response scales (e.g., yes, no, unsure). Table 1 presents all the questions of the questionnaire.

**Table 1:** Questionnaire

Q1	What is your age?
Q2	For how many years have you been playing men’s professional football?
Q3	Please state your nationality
Q4	Continental division you play in?
Q5	Do you play or have you previously played for a senior men’s (inter)national team?
Q6	Do you play for your team internationally?
Q7	What is the maximum number of back-to-back matches (across all competitions, including club and national team) that a player should play consecutively?
Q8	Do you feel that your off-season and/or in-season breaks are sometimes infringed upon by your club and/or national team?
Q9	Do you feel that an additional regulation or enforcement mechanism would be helpful in order to protect your break periods and prevent significant infringement upon them?
Q10	Do you believe the voice of players is respected and that the well-being of players is considered by those who develop and regulate the calendar and competition schedules?
Q11	During your career, do you believe you have suffered an injury as a result of a condensed or overloaded schedule?
Q12	Have you encountered any effect on your mental health and well-being with excessive workload demands or insufficient recovery periods being significant contributing factors?
Q13	Would you be in favour of longer but fewer international windows?
Q14	How often should the FIFA World Cup Finals take place?
Q15	How many national teams should qualify to play at the FIFA World Cup?
Q16	How would you describe the level of collaboration between your club and the national team staff in managing your playing and training loads?

### Procedures

An electronic survey (Typeform Professional) available in English, French, Italian and Spanish was compiled. Information about the study was given to potential participants by FIFPRO (email or verbally). If interested in the study, all participants gave their informed consent and completed the survey (coded and depersonalised). Data were collected in November 2021. Players participated voluntarily and did not receive any reward for their participation.

### Statistical analyses

The statistical software IBM SPSS 28.0 for Windows was used to perform all data analyses. Descriptive analyses (mean, standard deviation, frequency and/or range) were performed for all variables.

## Results

**Fig. 1:**
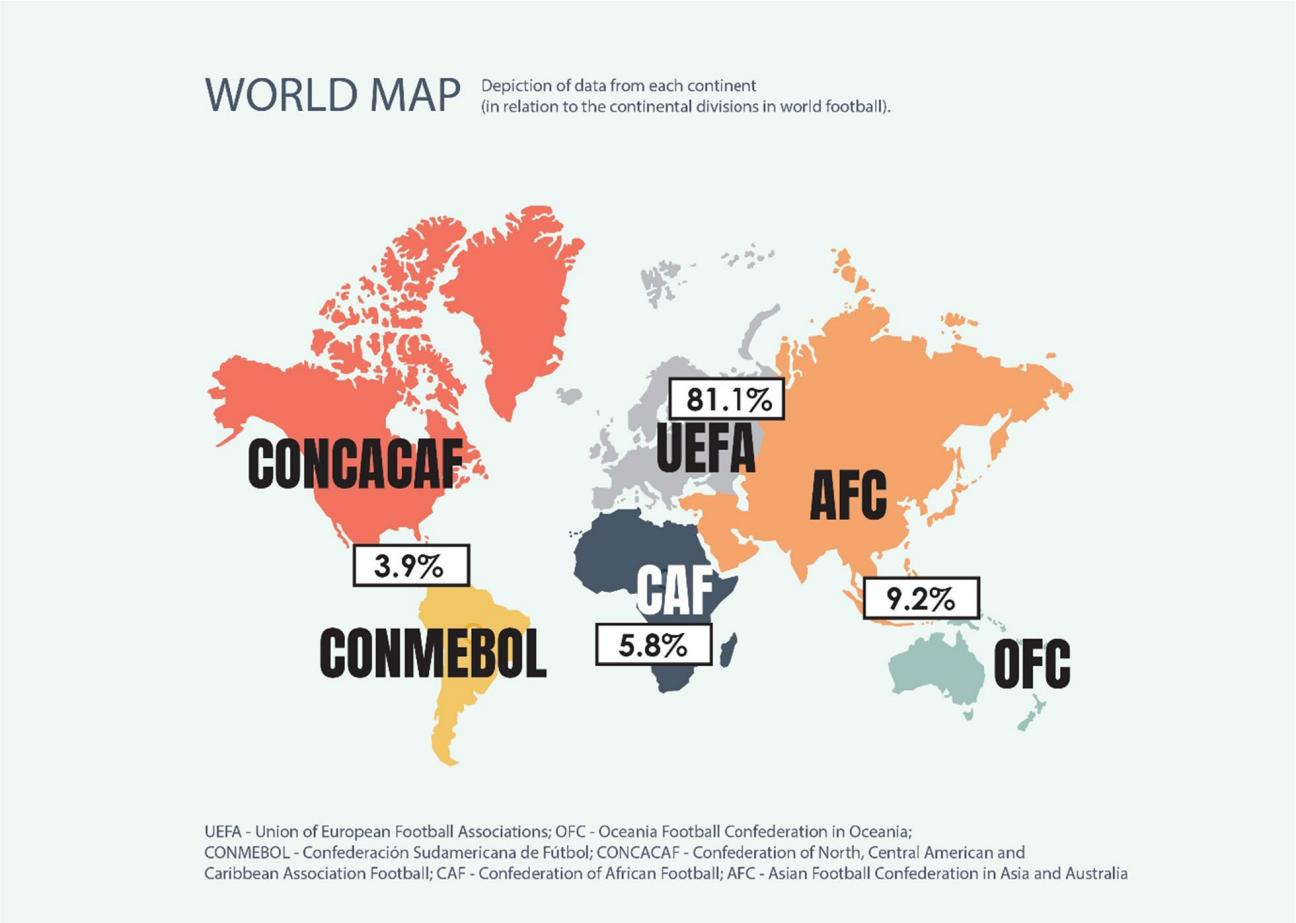
Continental divisions who answered questionnaires

A total of 1055 responses was received from players in 75 countries with Europe accounting for 81.1%, Asia and Oceania 9.2%, Africa 5.8% and the Americas 3.9% (Fig. 1).

**Fig. 2:**
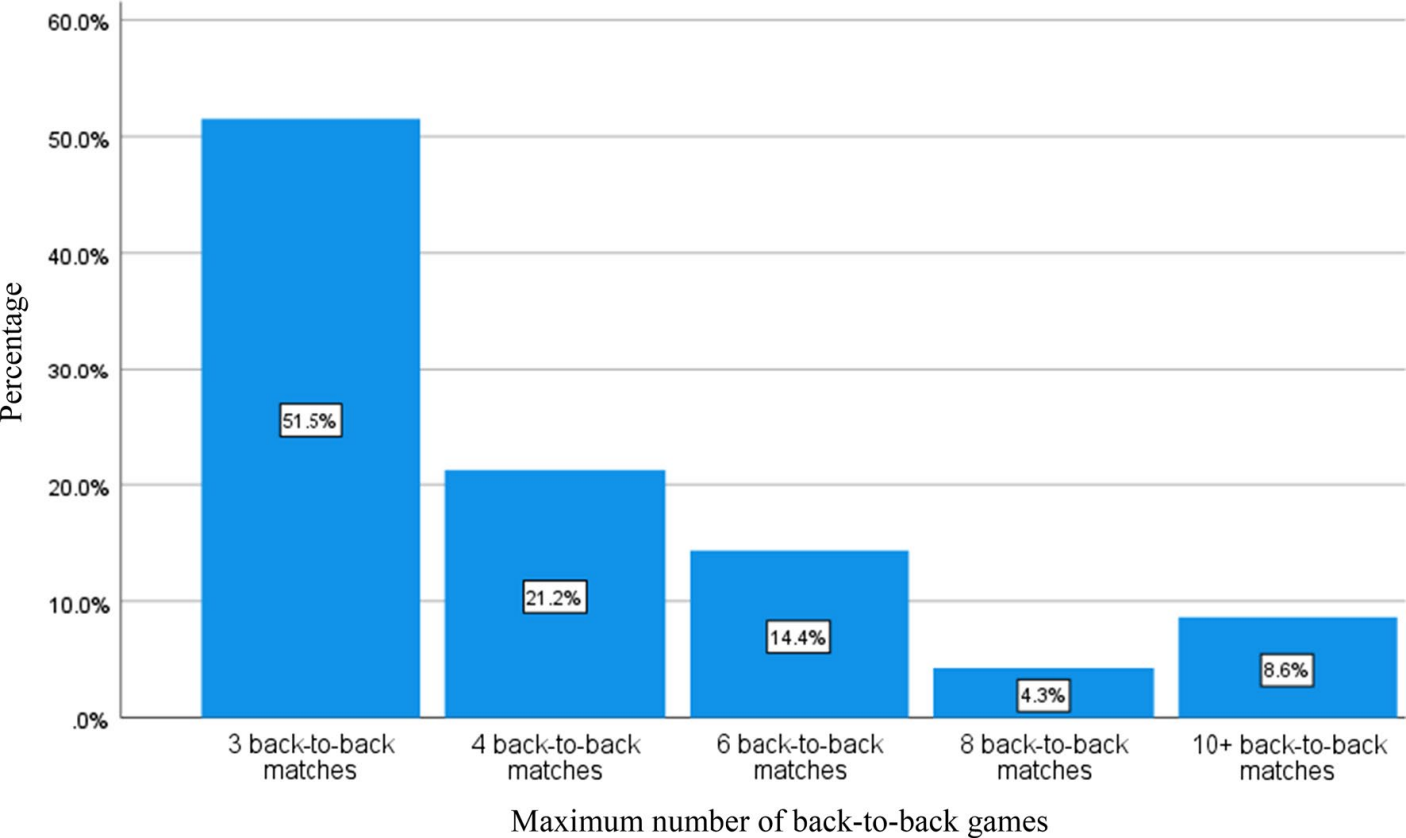
Maximum number of back-to-back games

The mean age was26.7 years, and professional playing years was 7.66 years. The majority of respondents ( 67.3%) never played for their National teams. Presently, 17.6% played for their National teams and 15.1% played in the past. Only 97.7% (n = 1031) of respondents answered the question regarding back-to-back game scheduling (n = 24 did not answer for unknown reasons). Half (50.3%) of the respondents felt that three back-to-back matches were the maximum amount of matches that should be allowed (Fig. 2).

As tabulated in Table 2, half of the respondents (50%) agreed that their off-season time was infringed upon by the club or country, while 76% believed there should be regulations to allow sufficient rest. Forty-eight percent (48%) of respondents believed that administrators deciding on the IMC do not consider their health and well-being when planning the IMC.

**Table 2:** Break infringements, regulations and players voices

	Yes	No	Unsure
Off/in-season breaks infringed by club/country	N = 529 (50.1%)	N = 336 (31.8%)	N = 190 (18%)
Regulations to prevent break infringements	N = 802 (76%)	N = 77 (7.3%)	N = 176 (16.7%)
Players voices respected in IMC decisions	N = 228 (21.6%)	N = 501 (47.5%)	N = 326 (30.9%)

Most respondents (76%) have indicated that they prefer a World Cup Final every four years instead of more frequent. Respondents (69%) also indicated that the traditional 32 teams for the World Cup were preferred. Communication between club and country indicated a high level in only 26% of respondents, while 14% noted communication sometimes (Table 3).

**Table 3:** International windows, World Cup frequency and number of teams, club/country collaboration

	Yes	No	Unsure	
Longer but fewer international windows	N = 490 (46.4%)	N = 271 (25.7%)	N = 292 (27.7%)	2 missing
	Every 2 years	Every 3 years	Every 4 years	
World cup frequency	N = 151 (14.3%)	N = 106 (10%)	N = 798 (75.6%)	
	32 (traditional)	48 (future)		
Number of teams at the World Cup	N = 730 (69.2%)	N = 320 (30.3%)		5 missing
	High level	Some level	None	Not applicable
Collaboration – club and country load management	N = 144 (13.6%)	N = 275 (26.1%)	N = 117 (11.1%)	N = 519 (49.2%)

While 35% of respondents believe they have sustained one injury due to match congestion, 20% believe they have sustained multiple injuries. Mental health and recovery were affected in 46% of respondents, as reflected in Table 4.

**Table 4:** Injury and effect on mental health due to congested schedule

	Yes once	Yes multiple	No	Unsure
Injury due to a congested schedule	N = 365 (34.6%)	N = 208 (19.7%)	N = 385 (36.5%)	N = 97 (9.2%)
	Yes	No	Unsure	
Congested fixtures and less recovery time effect on Mental health	N = 488 (46.3%)	N = 394 (37.3%)	N = 173 (16.4%)	

## Discussion

The important findings of this study are the following: (1) 50% of respondents believed that three back-to-back matches were the maximum amount that one should play, (2) 50% of respondents felt that there in/off season breaks were infringed upon by club or country, (3) 76% believe there should be regulations in place to protect them from these infringements on their breaks, (4) 48% felt that their opinions are not taken into account when the IMC scheduling is done, (5) 35% of respondents believe they sustained one injury due to match congestion while 20% believe they sustained multiple injuries due to match congestion, (7) 46% felt that due to match congestion and lack of recovery time, there mental health was negatively affected, (8) 46% preferred fewer but more extended international window periods, (9) 76% still prefer the traditional World Cup every 4 years, (10) 69% of respondents preferred the traditional 32 team World Cup squads, (11) only 14% felt that there was collaboration on load management between club and country medical staff.

A large proportion of respondents felt that fatigue from the lack of recovery time, their mental health, cognitive function and mood were negatively affected due to the congestion of the IMC. This study builds upon the available literature findings where players believe the congested fixture schedule has contributed to sustaining injuries and missing out on game time [6, 7, 9, 11, 13, 14, 16, 17]. However, the relationship between acute fatigue and cognitive performance needs further investigation in the literature [12, 18]. Half of the respondents in this study believed that three back-to-back games should be the maximum allowed. Other sports have shown a correlation between congested match schedules and injuries. The National Basketball Association (NBA) has shown a strong association between injuries, back-to-back games and away games (Gouttebarge et al., in 2019, investigated the negative effect on travel). Attention to the game schedule may reduce the incidence of injuries [19]. Field hockey (a multi-directional movement sport) has also shown a significantly increased risk of injury when scheduling games between twenty-four hours and three days after a fixture [20]. The football industry should be wise to realise these lessons from other sports, as fixture congestion may lead to a pending injury crisis within the international football environment.

While FIFA has recently strongly considered having a World Cup every two years, most respondents believe the current 4-year schedule is sufficient. A more regular World Cup competition (taking into account international qualifiers, travel and team match obligations) in an already congested IMC would certainly risk players from a mental, cognitive, injury, fatigue and performance perspective [16–18]. This probably explains why most respondents in this study believe they need to be protected from a regulation perspective to protect their health and well-being so they may continue to perform at optimal levels. Players may feel despondent, as half of the respondents believe their voices are not heard in IMC scheduling. This may further contribute to the negative effect on mental and performance health. A reduction in match congestion may lead to a lower incidence of injury, fatigue, cognitive and mental health effects, thereby maintaining performance health.

The upcoming FIFA World Cup in Qatar is also occurring at an unusual time in November 2022, i.e. midseason. This period is usually winter in the northern hemisphere and summer in the southern hemisphere. During this period, injury and mental health surveillance may reveal unusual patterns due to the midseason timing (effect on acute:chronic load) and prevailing weather conditions.

Communication of load monitoring when transitioning between club and country is vital to reduce the incidence of injury and maintain performance at both levels of play [21]. Respondents in the study confirm poor load monitoring communication between club and country medical staff.

The clinical relevance confirms and builds upon previous studies, suggesting that if players do not have optimal recovery time, it may affect them physically and mentally, exposing them to a higher rate of injuries and possibly mental health symptoms. This study expands on Gouttebarge et al.’s study by: (1) including players' opinions over more continental divisions with a larger cohort, (2) exploring ideal match load, (3) determining the acceptable frequency of World Cup events, (4) differentiating the negative impact of match congestion in relation to the effect on injury and mental health, (5) investigating communication between club and country regarding load and (6) acquiring players opinions regarding regulations and decision making in the IMC scheduling. A greater injury incidence would then mean that there will be less player availability which may affect team performance. Consequently, teams must have larger squads to allow for squad rotation. In light of high training and match loads, short recovery periods, and more possible injuries, teams will be spending extra financial resources on managing these physical injuries and any psychological issues.

Load monitoring communication between the club and country needs further exploration further. The authors propose a call for developing a standard template describing loads prior to National call-ups and upon return from National duty. Such a tool can assist in reducing injury incidence in light of a congested IMC. Repeating the questionnaire amongst the same cohort in future will be able to evaluate whether their opinions remain the same or change over time (taking the present IMC into perspective with uncertainty as to future IMC changes).

One of the limitations of this study is that more than half of the respondents have never played for their national team, so their opinion and perspective of some questions would only be from their observations of others and hear-say. However, they possibly play for teams competing in competitions dictated by the IMC. Another possible limitation, specifically regarding the number of respondents, may be that the questionnaires were distributed during a challenging part of the season. At this time, most leagues were moving toward the mid-season break. Players might feel pressured to perform on the field and not complete the questionnaires as the matches received their sole attention. Another limitation is that players may have had a hidden agenda regarding the IMC and saw this opportunity for FIFA to hear their voices since the majority felt their voices did not count. The purpose of this study was not to collect injury incidence or to determine injury rates (injuries/1000 h). Nevertheless, the data shows that players believe match congestion contributed to the increased rate of sustaining injuries.

## Conclusion

According to professional male players, the current IMC poses a great risk to their physical and mental health. The inability to recover physically and mentally between matches may eventually affect player availability and performance. Players should undoubtedly have actively playing players to represent them in decisions on IMC, enabling administrators to understand the burden and dangers they are exposing players to regarding IMC congestion (ideally done through a players representative association like FIFPRO). It may be worthwhile for football administrators to seek medical/scientific opinions regarding the effects of overload on performance before making IMC scheduling decisions. This study allows the opportunity for a larger National team representative player sample to be studied and the possible development of a load monitoring template to be shared between club and country.

## Data Availability

All available data is presented in the manuscript.

## Abbreviations

IMC: International Match Calendar
FIFA: Federation Internationale de Football Association World Cup
UEFA: Union of European Football Associations
FIFPRO: Fédération Internationale des Associations de Footballeurs Professionnels
COVID-19: Coronavirus
CAF: Confederation of African Football
